# Simultaneous Separation of Antioxidants and Carbohydrates From Food Wastes Using Aqueous Biphasic Systems Formed by Cholinium-Derived Ionic Liquids

**DOI:** 10.3389/fchem.2019.00459

**Published:** 2019-06-27

**Authors:** Catarina M. S. S. Neves, Marcos Figueiredo, Patrícia M. Reis, Ana C. A. Sousa, Ana C. Cristóvão, Mariana B. Fiadeiro, Luís Paulo N. Rebelo, João A. P. Coutinho, José M. S. S. Esperança, Mara G. Freire

**Affiliations:** ^1^Department of Chemistry, CICECO – Aveiro Institute of Materials, University of Aveiro, Aveiro, Portugal; ^2^LAQV/REQUIMTE, FCT-NOVA, Costa da Caparica, Portugal; ^3^CICS-UBI – Health Sciences Research Centre, University of Beira Interior, Covilhã, Portugal

**Keywords:** value added compounds, aqueous biphasic systems, circular economy, food waste, ionic liquids, resource efficiency, toxicity, waste valorization

## Abstract

The food industry produces significant amounts of waste, many of them rich in valuable compounds that could be recovered and reused in the framework of circular economy. The development of sustainable and cost-effective technologies to recover these value added compounds will contribute to a significant decrease of the environmental footprint and economic burden of this industry sector. Accordingly, in this work, aqueous biphasic systems (ABS) composed of cholinium-derived bistriflimide ionic liquids (ILs) and carbohydrates were investigated as an alternative process to simultaneously separate and recover antioxidants and carbohydrates from food waste. Aiming at improving the biocompatible character of the studied ILs and proposed process, cholinium-derived bistriflimide ILs were chosen, which were properly designed by playing with the cation alkyl side chain and the number of functional groups attached to the cation to be able to create ABS with carbohydrates. These ILs were characterized by cytotoxicity assays toward human intestinal epithelial cells (Caco-2 cell line), demonstrating to have a significantly lower toxicity than other well-known and commonly used fluorinated ILs. The capability of these ILs to form ABS with a series of carbohydrates, namely monosaccharides, disaccharides and polyols, was then appraised by the determination of the respective ternary liquid-liquid phase diagrams at 25°C. The studied ABS were finally used to separate carbohydrates and antioxidants from real food waste samples, using an expired vanilla pudding as an example. With the studied systems, the separation of the two products occurs in one-step, where carbohydrates are enriched in the carbohydrate-rich phase and antioxidants are mainly present in the IL-rich phase. Extraction efficiencies of carbohydrates ranging between 89 and 92% to the carbohydrate-rich phase, and antioxidant relative activities ranging between 65 and 75% in the IL-rich phase were obtained. Furthermore, antioxidants from the IL-rich phase were recovered by solid-phase extraction, and the IL was recycled for two more times with no losses on the ABS separation performance. Overall, the obtained results show that the investigated ABS are promising platforms to simultaneously separate carbohydrates and antioxidants from real food waste samples, and could be used in further related applications foreseeing industrial food waste valorization.

## Introduction

According to the Food and Agriculture Organization of the United Nations (FAO), one third of the food worldwide produced for human consumption (1.3 billion tons per year) is lost or wasted (FAO, 2011). In Europe, *ca*. 88 million tons of food waste are generated *per* year, with associated costs of 143 billion euros (Fusions, 2016). Currently, food waste constitutes a relevant economic problem and is linked to negative societal and environmental impacts (Fusions, 2016), being responsible for about 8% of the Global Greenhouse Gas emissions (European Commission, 2018). As a result, the management of food waste, including its reuse, is a priority mitigation measure to reduce emissions intensity and the carbon footprint of the food production chain (IPCC, 2018). According to the Intergovernmental Panel on Climate Change (IPCC) this can be achieved through new technological solutions, e.g., by transforming food waste into products with marketable value (IPCC, 2018). These new solutions will not only reduce the environmental impact of the food industry, but will also contribute to improve its economic impact as the residues generated may contain value added compounds. Furthermore, sustainable food systems have a pivotal role in the UN Sustainable Developmental Goals (European Commission, 2018; Pradyumna, 2018). Overall, and in the framework of circular economy, these facts stress the need to develop sustainable and cost-effective technologies to recover value added compounds from food waste, aiming at contributing to a decrease of the environmental footprint and increased sustainability of this industrial sector.

Several extraction methods are currently applied for the valorization of food waste, including hydrothermal extraction, supercritical fluid extraction, pressurized liquid extraction, microwave-assisted extraction, and ultrasound-assisted extraction (see Arshadi et al., 2016 and references therein). However, some of these technologies display several drawbacks, including high capital investment and/or high operating costs, as well as potential negative environmental impacts associated to high-energy consumption and/or by the use of volatile organic solvents. In order to avoid the use of those and to decrease the economic burden, an increasing interest on the application of ionic liquids (ILs) as alternative solvents in the food industry sector has emerged (Toledo Hijo et al., 2016). Ionic liquids properties, including their ability to be tailored in terms of physicochemical properties and designed to a specific application through the manipulation of their ions (Rogers and Seddon, 2003; Ventura et al., 2017), make them ideal candidates to be used in the valorization of food waste. Ionic liquids not only have the ability to dissolve and pretreat complex raw materials, but they can be also designed to selectively extract target compounds due to their tailoring ability. Nonetheless, when compared with their use in the extraction of value added products from biomass (Passos et al., 2014), few studies addressed the ILs application in the recovery of value added compounds from real food waste (Lateef et al., 2009; Bi et al., 2010; Qin et al., 2010; Bica et al., 2011; Guolin et al., 2012; Setoguchi et al., 2012; Cláudio et al., 2013, 2018; Jiao et al., 2013; Ge et al., 2014; Hernoux-Villière et al., 2014; Zhang et al., 2015, 2017; de Faria et al., 2017; Oberleitner et al., 2017; Mizuno and Usuki, 2018). Most of these studies employed imidazolium-based ILs to recover antioxidants, vitamins, fats, sugars, and essential oils from different types of food and food waste, such as tea, fruits, vegetables, crustaceans, or used oils. However, given the often moderate to high toxicity and poor biodegradability of imidazolium-based ILs, the application of novel and more biocompatible ILs, such as cholinium-based, started to emerge (Garcia et al., 2010; Ni et al., 2012; Wang et al., 2016; Oberleitner et al., 2017).

ILs can be used to form aqueous biphasic systems (ABS), which due to their water-rich media can be applied in sustainable and biocompatible separation processes. Most IL-based ABS are ternary systems formed by water, one IL and a third species with salting-out capacity, such as an inorganic salt with high-charge density (Freire et al., 2012). More recently, novel combinations to create IL-based ABS have been proposed by combining ILs with amino acids, polymers, or carbohydrates in aqueous media (Ventura et al., 2017). In addition to the characterization of their phase diagrams, a plethora of systems has been investigated in the extraction and separation of value added compounds from aqueous media (Freire et al., 2012). However, most of these studies were carried out with model systems, and the use of IL-based ABS for the recovery of target compounds from real matrices has seldom been studied (Ventura et al., 2017).

Although a large number of works on IL-based ABS have been reported (Freire et al., 2012; Ventura et al., 2017; Song et al., 2018; Yee et al., 2018), few works on the formation of IL-based ABS by the addition of carbohydrates are available since more hydrophobic (yet water-soluble) ILs are required (Zhang et al., 2007; Wu et al., 2008a,b,c; Chen et al., 2009, 2010, 2012; Chen and Zhang, 2010; Freire et al., 2011; Ferreira et al., 2016; Okuniewski et al., 2016; Quental et al., 2018). This trend is due to the weak salting-out aptitude of carbohydrates when compared to high-charge density salts. Therefore, imidazolium-based cations combined with tetrafluoroborate and triflate anions have been the most studied ILs (Freire et al., 2012). Nevertheless, these ILs may display some citotoxicity concerns, as summarized in Table 1. In this work, we demonstrate that less cytotoxic cholinium-derived ILs can form ABS with carbohydrates, achieved by a proper tailoring of the IL anion and cation alkyl side chain and number of attached functional groups. Taking into account the possibility of creating ABS with carbohydrates and more biocompatible cholinium-derived ILs, here established by cytotoxicity assays toward human intestinal epithelial cells (Caco-2 cell line), these systems may be anticipated as improved strategies to separate carbohydrates and antioxidants from food wastes.

**Table 1:** Cytotoxicity of ILs containing fluorinated anions toward the Caco-2 cell line evaluated through the MTT [3-(4,5-dimethylthiazol-2-yl)-2,5-diphenyltetrazolium bromide] assay.

**Ionic liquid**	**Objective/Application**	**EC_**50**_ (mM)**	**References**
[C_4_C_1_im][BF_4_]	To compare the cytotoxicity of amino-acid- and imidazolium-based ILs	11.19 (±0.63)^a^	Egorova et al., 2015a
[C_4_C_1_im][PF_6_]		11.50 (±1.08)^a^	Egorova et al., 2015a
[Ala-OMe][BF_4_]	To address the citotoxicity of new drug delivery platforms based on imidazolium-based ILs	6.24 (4.32–8.17)^b^	Egorova et al., 2015b
[C_2_C_1_im-OSal][BF_4_]		4.77 (3.27–6.27)^b^	Egorova et al., 2015b
[C_8_C_1_im][PF_6_]	To evaluate the cytotoxicity of imidazolium-based ILs and application of models	5.12 (±31.81)^c^	García-Lorenzo et al., 2008
[C_6_C_1_im][PF_6_]		15.67 (±3.78)^c^	García-Lorenzo et al., 2008

*Only studies with a 24 h exposure period are provided in order to allow direct comparisons with our results*.

a *Standard error of the mean (SEM)*.

b *95% Confidence interval*.

c *Standard deviation (SD)*.

In ABS formed by ILs and carbohydrates there is the spontaneous separation of the two-phase forming components above given concentrations, whereas previous works on ABS formed by ILs and salts support the preferential migration of antioxidants to the IL-rich phase (Cláudio et al., 2010, 2012, 2014). These patterns lead us to envision that both products (antioxidants and carbohydrates) may be enriched in separated phases in ABS formed by ILs and carbohydrates. This hypothesis was here validated under a perspective of resource efficiency and circular economy, in which ABS composed of cholinium-derived ILs and carbohydrates were investigated to simultaneously separate antioxidants and carbohydrates from food waste, namely from an expired commercial vanillin-rich pudding.

## Materials and Methods

### Materials

**Figure 1:**
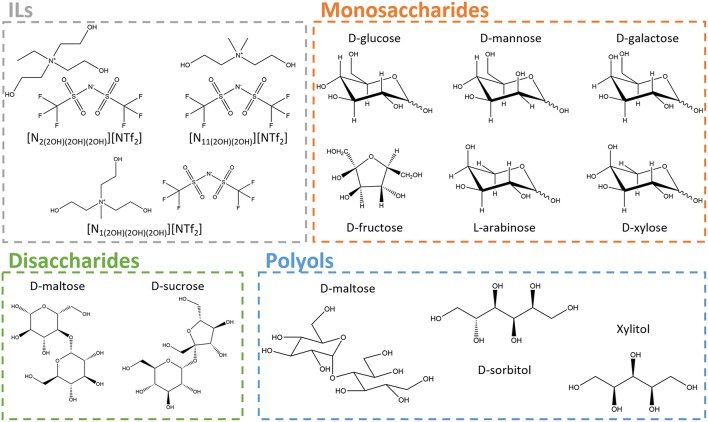
Chemical structures of ionic liquids (ILs) and different classes of carbohydrates (monosaccharides, disaccharides, and polyols) studied.

The chemical structures of the ILs used in this work [*N*-methyl-*N,N,N*-tris(2-hydroxyethyl)ammonium bistriflimide, [N_1(2OH)__(2OH)__(2OH)_][NTf_2_]; *N*-ethyl-*N,N,N*-tris(2-hydroxyethyl)ammoniumbistriflimide,[N_2(2OH)(2*OH*)__(2*OH*)_][NTf_2_]; *N, N*-dimethyl-*N,N*-bis(2-hydroxyethyl) ammonium bistriflimide, [N_11(2OH)__(2OH)_][NTf_2_]] are presented in Figure 1. The ILs investigated were synthesized according to the protocol described by de Ferro et al. (2018). The AgNO_3_ test was performed to all ILs to confirm the absence of halides (chloride or bromide). Characterization analysis by ^1^H and ^19^F NMR and Elemental Analysis indicate that all ILs have a purity >99 wt%. These results are given in the Supplementary Material. Prior to their use, all ILs were dried for a minimum of 48 h at 35°C under vacuum (≈0.1 Pa) with constant stirring. The water content of the dried ILs, measured by coulometric Karl Fischer titration, was below 500 ppm. Carbohydrates used in this work comprise monosaccharides, disaccharides and polyols, namely: D-maltitol (≥95 wt%) and xylitol (≥99 wt%) acquired from Acros Organics; D-(+)-maltose (99 wt%) acquired from BDH; D-sorbitol (≥91 wt%) from Fisher BioReagents; D-(+)-sucrose (≥99 wt%) and D-(+)-galactose (≥98 wt%) from GPR Rectapur; D-(+)-glucose (99 wt%) from Scharlau; D-(-)-fructose (≥99 wt%) and L-(+)-arabinose (≥98 wt%) from PanReac AppliChem; D-(+)-mannose (≥99 wt%) from Alfa Aesar; and D-(+)-xylose (≥99 wt%) from Merck. The chemical structures of the investigated carbohydrates are depicted in Figure 1.

The water used was ultra-pure, double distilled, passed by a reverse osmosis system and further treated with a Milli-Q plus 185 water purification apparatus. For the antioxidant assays, the radical 2,2-diphenyl-1-picrylhydrazyl (DPPH) obtained from Sigma Aldrich was used. The Dubois method (Dubois et al., 1956) was used to determine the total content of carbohydrates in each phase, for which sulfuric acid (95 wt%) from Sigma-Aldrich and phenol (99.5 wt%) from Merck were used. A commercial vanillin-rich pudding from a Portuguese brand was used at a concentration of 18 wt% in water as the food waste sample. An Oasis HLB (200 mg) SPE cartridge from Waters was used in the solid-phase extractions to separate the IL from carbohydrates and antioxidants. Methanol of HPLC grade, acquired from Sigma-Aldrich, was used.

### Cytotoxicity Assays

The cytotoxicity of each IL was evaluated in the human colon carcinoma cell line (Caco-2) using the 3-(4,5-dimethylthiazol-2-yl)-2,5-diphenyltetrazolium bromide (MTT) assay. The Caco-2 cell line was used since this cancer derived cell line is a well-established model of the intestinal epithelial barrier, being widely used in the toxicological evaluation of pharmaceutical drugs (Meunier et al., 1995) and new food bioactive compounds (Lea, 2015). The MTT assay is based on the ability of viable cells to convert MTT into formazan, a water insoluble compound that can be colorimetrically quantified. It is a widely used assay to evaluate cytotoxicity being suitable for high throughput screening (HTS) (Riss et al., 2016; Sharma et al., 2018; Macário et al., 2019; Thamke et al., 2019). Furthermore, the available published studies performed with the same cell line (Caco-2 cell line) and the same incubation period (24 h) used MTT (García-Lorenzo et al., 2008; Egorova et al., 2015a,b); therefore, this assay also allows to perform comparisons with other fluorinated ILs used in the creation of ABS with carbohydrates.

Cells were grown in high glucose Dulbecco's modified Eagle's medium (DMEM-HG) with 10% (v/v) fetal bovine serum (FBS), 100 units penicillin, and 50 μg·mL^−1^ streptomycin in a humidified atmosphere of 5% CO_2_ at 37°C. Different concentrations of ILs (0.01, 0.1, 1.0, 30, 60, and 90 g·L^−1^) were prepared in saline [[NaCl] = 0.9% (m/w)] aqueous solutions. For the toxicological assays, cells were plated on polystyrene cell culture dishes at a density of 1 × 10^4^ cells *per* well in 96 well culture plates. Upon 16 h after plating, cells were treated with different concentrations of the target ILs for 24 h. Afterwards, cell variability was assessed using the MTT assay. Caco-2 cells were incubated overnight at 37°C with 0.5 mg·L^−1^ of MTT, and the water-insoluble precipitate was dissolved in 10% (w/v) sodium dodecyl sulfate SDS for 4 h. The colorimetric detection was performed at 570 nm using a microplate spectrophotometer. Each concentration was tested in at least four replicates of three independent experiments (*n* = 3). The dose response curves and EC_50_ calculations were performed using the GraphPad PRISM Software (version 5.01). The average effective concentration (EC_50_), i.e., the concentration of IL at which 50% of cells are viable, was calculated using a sigmoidal dose response equation using the automatic outliers fitting method.

### ABS Phase Diagrams and Their Application as Simultaneous Separation Strategies of Antioxidants and Carbohydrates

The ternary phase diagrams were determined at room temperature (≈25°C) and at atmospheric pressure by the cloud point titration method (Freire et al., 2011). Stock solutions of each carbohydrate (ranging from 40 to 70 wt%, depending on the carbohydrate solubility in water) and of each IL (≈70 wt%) were prepared and used for the determination of the phase diagrams. Repetitive drop-wise addition of each carbohydrate solution to each IL aqueous solution was carried out until the detection of a cloudy solution, followed by the drop-wise addition of ultra-pure water until the detection of a monophasic region (clear and limpid solution). All these additions were carried out under continuous stirring. When no more cloud points were detected, an inversion of the method was applied, i.e., with the repetitive addition of the IL solution to the carbohydrate solution aiming at gathering more experimental data points to better define each binodal curve. In both cases, the systems composition was determined by weight (±10^−4^ g). Not all the combinations of ILs and carbohydrates investigated are able to form ABS—a complete list of the combinations tested is given in Supplementary Table S1.

The ABS formed by ILs and carbohydrates were then investigated to simultaneously separate antioxidants and carbohydrates from food waste. The food waste sample used corresponds to a vanilla pudding, thus rich in the antioxidant vanillin, of a Portuguese brand. The pudding was dissolved at room temperature (*ca*. 25°C) in distilled water for a minimum of 2 h under continuous agitation. This solution was then left for 20 min in order to allow the deposition of the non-water soluble solids, and further decanted. The obtained supernatant was centrifuged at 1,100 rpm for 10 min in an Eppendorf Centrifuge 5804, and the liquid fraction was filtered with PTFE membrane filters with a porosity of 0.45 μm. This aqueous solution, rich in both carbohydrates and antioxidants, was used in the ABS composition and formation. The mixture composition investigated corresponds to: 25 wt% D-glucose or D-sucrose + 50 wt% IL + 25 wt% aqueous solution of pudding. After mixing, each ABS was left overnight at 25°C, phases were separated, and the content of carbohydrates and antioxidants in each phase was quantitatively determined.

The content of antioxidants in each phase was appraised by the determination of the respective antioxidant activity using the DPPH radical scavenging assay, according to the method described by Sintra et al. (2015). Slight modifications were however introduced, in which methanol was substituted by ethanol in order to use more benign solvents. Briefly, 125 μL of DPPH solution (0.36 g·L^−1^ in ethanol) were mixed under dark conditions with each ABS phase (250 μL). Water was added up to 1 mL (final volume). All tubes were vortex mixed and kept for equilibrium in the dark for 30 min. Control samples were prepared and used. The absorbance of the samples was determined at 540 nm using a microplate spectrophotometer. Triplicate samples were prepared and the values given correspond to the average value and associated standard deviation.

The percentage antioxidant activity (%*AA*) was determined using Equation (1):

(1) $$\begin{array}{r} \%AA=\;\frac{A_{0}-A_{1}}{A_{0}}\times 100 \end{array}$$

where *A*_0_ is the control absorbance and *A*_1_ is the absorbance of the sample at the maximum wavelength.

The antioxidant relative activity (%*ARA*) in each ABS phase was calculated according to Equation (2):

(2) $$\begin{array}{r} \%ARA=\;\frac{AA_{phase}}{AA_{top}+AA_{bot}}\times 100 \end{array}$$

where *AA*_*phase*_ is the antioxidant activity of the respective ABS phase, and *AA*_*top*_ and *AA*_*bot*_ are the antioxidant activity of the ABS top and bottom phases, respectively.

The total carbohydrates quantification in each ABS phase was performed through a colorimetric assay based on the Dubois method (Dubois et al., 1956). The colored product was quantified at 488 nm using a microplate spectrophotometer. The calibration curve was prepared with D-glucose. Triplicate samples were prepared and the values given correspond to the average value and associated standard deviation. The extraction efficiency (%*EE*) of carbohydrates was determined as the ratio between the carbohydrates concentration in the respective ABS phase and the total concentration of carbohydrates in both phases, according to the Equation (3):

(3) $$\begin{array}{r} \%EE=\;\frac{{\left[CH\right]}_{phase}}{{\left[CH\right]}_{top}+{\left[CH\right]}_{bot}}\times 100 \end{array}$$

where [*CH*]_*phase*_ is the carbohydrates concentration in the ABS respective phase, and [*CH*]_*top*_ and [*CH*]_*bot*_ are the carbohydrates concentrations in the ABS top and bottom phases, respectively.

The separation of the IL from the antioxidants in the IL-rich phase was performed by solid-phase extraction, with Oasis HLB cartridges previously conditioned with methanol (1 mL) and equilibrated with acidic water (pH 2, 1 mL) (Waters, 2014). The IL-rich phase was loaded through the column, where carbohydrates and antioxidants are adsorbed, followed by the loading of 3 mL of acidic water to recover the IL. Then, 2 mL of a mixture of methanol/water (20:80, v:v) was used to desorb carbohydrates and antioxidants (Michalkiewicz et al., 2008). The IL purity after recovery was confirmed through ^1^H NMR, and the respective losses determined gravimetrically (±10^−4^ g). After the separation through SPE, the fractions containing the IL were subjected to evaporation under vacuum at 60°C. The recovered non-volatile IL was then reused in the creation of novel ternary systems. The IL recycling was tested for two times. The carbohydrates quantification and the antioxidant activity were determined by the methods described above.

## Results and Discussion

### ILs Cytotoxicity Analysis

In this work, we focused on the use of new cholinium-derived ILs with the bistriflimide anion, known to produce hydrophobic ILs, to create novel ABS with weak salting-out substances such as carbohydrates. We manipulated the chemical structure of the cholinium cation by grafting it with additional hydroxyethyl moieties to produce water soluble bistriflimide-based ILs. Complete water miscibility of the studied ILs (shown in Figure 1) is achieved due to the large number of OH groups at the cation, contributing to stronger hydrogen-bonding with water (de Ferro et al., 2018).

**Figure 2:**
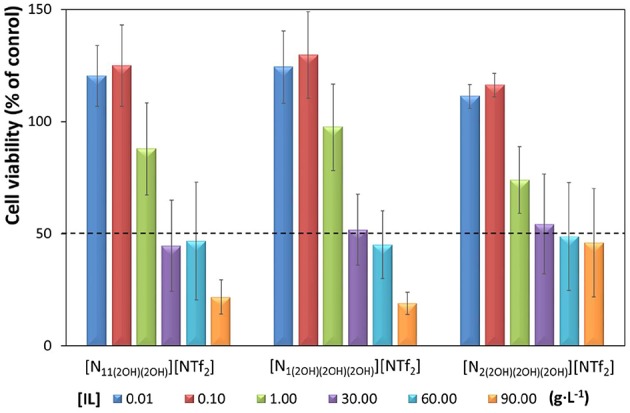
Caco-2 cell viability after 24 h of exposure to different concentrations of each IL. The experimental points, expressed as percentage of the control, correspond to the average of at least four replicates of three independent experiments (*n* = 3) and the lines correspond to respective standard error of the mean (SEM). The control corresponds to untreated cells. Values of cell viability higher than 100% are indicative of cell proliferation. The dash line indicates the EC_50_ values for each IL.

Given the hypothesis to be evaluated, i.e., if ABS formed by carbohydrates and ILs are platforms with potential to simultaneously separate carbohydrates and antioxidants from food waste, the evaluation of the ILs citotoxicity toward Caco-2 cells was here carried out as a first test to appraise their biocompatible nature. The cytotoxic profile of the investigated ILs toward the Caco-2 cell line is shown in Figure 2. The cytotoxic profile of [N_11(2OH)__(2OH)_][NTf_2_] and [N_1(2OH)__(2OH)__(2OH)_][NTf_2_] is similar, where an increase in the IL concentration leads to an increase in cytotoxicity. For both ILs, an increase in the cell viability (<30%) at the lowest concentrations tested (0.01 and 0.1 g·L^−1^) is observed; however, this increase is not statistically significant (*p* > 0.05). The EC_50_ and respective 95% confidence intervals (CI) values are also similar between these two ILs, with 29.19 g·L^−1^ (26.90–31.48 g·L^−1^) for [N_11(2OH)__(2OH)_][NTf_2_] and 29.48 g·L^−1^ (28.35–30.61 g·L^−1^) for [N_1(2OH)__(2OH)__(2OH)_][NTf_2_]. For [N_2(2OH)__(2OH)__(2OH)_][NTf_2_] a different cytotoxic pattern is seen. For this IL, concentrations higher than 30 g·L^−1^ do not lead to an increased toxicity. About 50% of viable cells are present for the higher concentrations investigated, namely 30, 60, and 90 g·L^−1^, and for this reason it is not possible to calculate the EC_50_ value for this particular IL (*cf*. Supplementary Figure S1). However, this trend means that this IL exhibits low toxicity in the tested concentrations, and at least up to 90 g·L^−1^. Overall, taking into account the EC_50_ values and respective standard errors shown in Figure 2, it is safe to admit that [N_2(2OH)__(2OH)__(2OH)_][NTf_2_] has a similar cytotoxic impact when compared to the remaining ILs studied.

The comparison of the cytotoxic impact of the investigated ILs and of other ILs reported in the literature, whose data were obtained under the same experimental conditions (Caco-2 cell line and 24h of exposure period), is given in Table 1 and Supplementary Figure S2. Ionic liquids comprising cations such as imidazolium and alanine derivatives combined with fluorinated anions are significantly more toxic than the cholinium-derived bistriflimide ILs investigated in this work. By combining the [NTf_2_]^−^ anion with cholinium derivatives cations, it is possible to reduce their average toxicity by *ca*. 10 times when comparing with the ILs O-methylalaninate tetrafluoroborate ([Ala-OMe][BF_4_]), 1-(2-((2-hydroxybenzoyl)oxy)ethyl)-3-methylimidazolium tetrafluoroborate ([C_2_C_1_im-OSal][BF_4_]) and 1-methyl-3-octylimidazolium hexafluorophosphate ([C_8_C_1_im][PF_6_]) (Egorova et al., 2015a,b), and *ca*. 4 times when comparing with 1-hexyl-3-methylimidazolium hexafluorophosphate ([C_6_C_1_im][PF_6_]) (García-Lorenzo et al., 2008). Such results demonstrate that it is possible to design new ILs based on the cholinium cation and fluorinated anions, such as bistriflimide, that are significantly less toxic than other fluorinated-based ILs commonly used. Despite such promising results, it must be remarked that a complete risk assessment of these ILs always needs to be performed before their safety can be completely established. This evaluation should include more *in vitro* tests with other cell lines and different endpoints, as well as *in vivo* experiments with organisms from different taxa (including e.g., invertebrates and mammals).

### ABS Phase Diagrams

**Figure 3:**
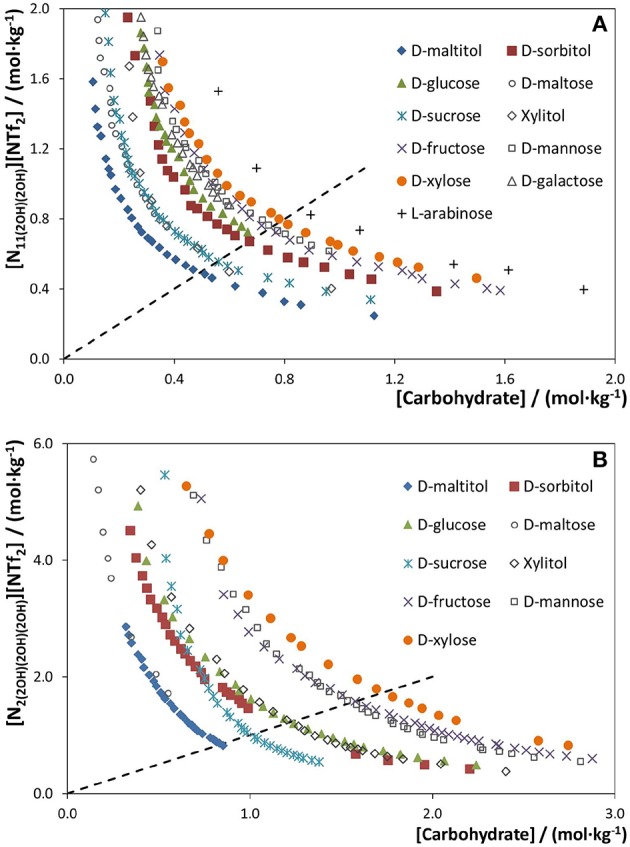
Phase diagrams of ABS composed of water, different carbohydrates and ILs, at 25°C and atmospheric pressure, to address the carbohydrate effect: **(A)** [N11(2OH)(2OH)][NTf2] and **(B)** [N2(2OH)(2OH)(2OH)][NTf2]. The dashed line represents [IL] = [Carbohydrate].

The phase diagrams of each ternary system formed by water, IL and carbohydrate were determined at atmospheric pressure and 25°C. Three different classes of carbohydrates were tested to promote phase separation, namely monosaccharides (D-glucose, D-mannose, D-galactose, D-fructose, L-arabinose, and D-xylose), disaccharides (D-maltose and D-sucrose), and polyols (D-maltitol, D-sorbitol, and xylitol), combined with three ILs, namely [N_1(2OH)__(2OH)2OH)_][NTf_2_], [N_2(2OH)__(2OH)2OH)_][NTf_2_], and [N_11(2OH)2OH)_][NTf_2_]. The chemical structures of the investigated ILs and carbohydrates are given in Figure 1. Figure 3 depicts the binodal curves in molality units of each ABS determined by the cloud point titration method (Freire et al., 2011). Compositions of IL and carbohydrate above each binodal curve result in two-phase systems, whereas mixture compositions below fall within the monophasic region. In summary, a larger biphasic region corresponds to a higher ability of both the carbohydrate and the IL to induce phase separation, thus requiring lower amounts of these phase-forming components to create ABS. The detailed data and respective ternary phase diagrams in weight fraction are provided in Supplementary Figures S3, S4 and Supplementary Tables S2–S4. Not all combinations of ILs and carbohydrates are able to form ABS at the studied conditions—a list of the successful combinations is provided in Supplementary Table S1.

Although all carbohydrates were tested with the three ILs, only the most hydrophobic, i.e., [N_11(2OH)__(2OH)_][NTf_2_], with a lower number of hydroxyl groups is able to form ABS with all the studied carbohydrates. This is an indication of the carbohydrates poor salting-out potential over ILs, in agreement with the literature (Freire et al., 2011). The lower the IL affinity for water, here achieved by reducing the number of hydroxyl groups and propensity for hydrogen-bonding with water, the easier it is to induce its salting-out and to create ABS. The general trend of the carbohydrates ability to salt-out the IL or to create ABS with [N_11(2OH)__(2OH)_][NTf_2_] shown in Figure 3A, obtained when the molality of IL equals the molality of carbohydrate in the binodal curve ([IL] = [Carbohydrate], in mol·kg^−1^), follows the order: D-maltitol > D-maltose ≈ D-sucrose ≈ xylitol > D-sorbitol > D-glucose > D-galactose > D-mannose ≈ D-fructose > D-xylose > L-arabinose. This trend is in good agreement to those previously reported in the literature (Freire et al., 2011), demonstrating that polyols are more effective than saccharides at promoting phase separation. Furthermore, it is shown that 6-sided ring carbohydrates are stronger salting-out agents than the 5-sided ring ones, as well as carbohydrates with a higher number of hydroxyl groups (increasing thus the propensity to hydrogen bond with water), being these more effective in the creation of ABS with ILs. A deeper discussion on these effects can be found in the literature (Freire et al., 2011; Ferreira et al., 2016). On the other hand, when the hydrophilicity of the IL is increased by the replacement of a methyl group in the cation by a hydroxyethyl group, the number of carbohydrates able to promote phase separation decreases. This trend is shown in Figure 3B for the second most hydrophobic IL investigated, [N_2(2OH)__(2OH)__(2OH)_][NTf_2_]. The binodal curves corresponding to the more hydrophilic IL [N_1(2OH)__(2OH)__(2OH)_][NTf_2_] are given in Supplementary Figure S4. The carbohydrates rank to form ABS with these ILs is as follows: D-maltitol ≈ D-maltose > D-sucrose > D-sorbitol > D-glucose ≈ xylitol > D-mannose ≈ D-fructose > D-xylose, being in close agreement with the previous one and to what has been found with other fluorinated ILs (Ferreira et al., 2016).

**Figure 4:**
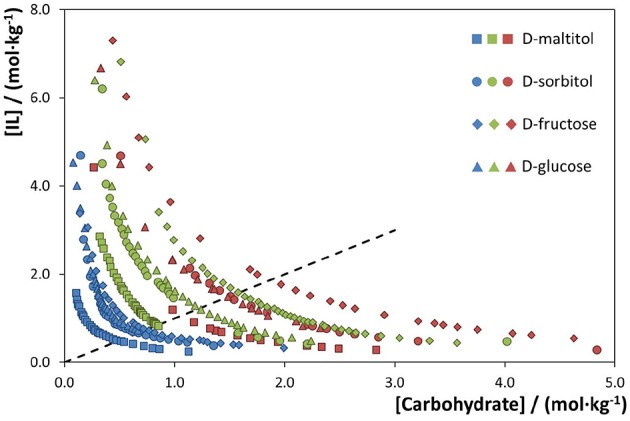
Phase diagrams of ABS composed of water and different carbohydrates and ILs, at 25°C and atmospheric pressure, to address the IL effect. Red symbols correspond to the IL [N_1(2OH)__(2OH)__(2OH)_][NTf_2_], green symbols to [N_2(2OH)__(2OH)__(2OH)_][NTf_2_], and blue symbols to [N_11(2OH)__(2OH)_][NTf_2_]. The dashed line represents [IL] = [Carbohydrate].

The binodal curves of ABS composed of different ILs and a fixed carbohydrate are provided in Figure 4. Examples with four carbohydrates are given. With a common carbohydrate, the IL ability to create ABS follows the order: [N_11(2OH)__(2OH)_][NTf_2_] > [N_2(2OH)__(2OH)__(2OH)_][NTf_2_] > [N_1(2OH)__(2OH)__(2OH)_][NTf_2_]. The phase diagrams corresponding to a common carbohydrate and the most hydrophobic IL ([N_11(2OH)__(2OH)_][NTf_2_]) are the ones with the largest biphasic regions, whereas those corresponding to the ILs [N_2(2OH)__(2OH)__(2OH)_][NTf_2_] and [N_1(2OH)__(2OH)__(2OH)_][NTf_2_] display smaller biphasic regions. In general, an increase in the cation alkyl side chain from methyl to ethyl, i.e., from [N_1(2OH)__(2OH)__(2OH)_][NTf_2_] to [N_2(2OH)__(2OH)__(2OH)_][NTf_2_], increases the IL hydrophobicity and thus its ability to be salted-out by the carbohydrate and to create ABS. Moreover, when analyzing the overall trend, it is demonstrated that the number of hydroxyl groups at the IL cation has a stronger effect on phase separation in ABS than the effect of increasing the cation alkyl side chain length (from methyl to ethyl). ILs with a lower number of hydroxyl groups are more hydrophobic and more easily salted-out by each carbohydrate.

A comparison of ABS with other fluorinated ILs [1-butyl-3-methylimidazolium triflate, ([C_4_C_1_im][CF_3_SO_3_]) (Freire et al., 2011), 1-butyl-3-methylimidazolium tetrafluoroborate ([C_4_C_1_im][BF_4_]) (Freire et al., 2011), and 1-ethyl-3- methylpyridinium perfluorobutanesulfonate [C_2_C_1_py][C_4_F_9_SO_3_] (Ferreira et al., 2016)] and D-glucose is given in Supplementary Figure S6. Amongst all the studied ILs as phase-forming components of ABS with carbohydrates, it is shown that the IL [N_11(2OH)__(2OH)_][NTf_2_] is the one that requires a lower amount of carbohydrates to undergo phase separation. This fact is relevant from an application point of view since lower quantities of IL and carbohydrate are required to form ABS, while reinforcing their higher biocompatible character due to their higher water content. On the other hand, the IL [N_1(2OH)__(2OH)__(2OH)_][NTf_2_] is the one that requires higher amounts of IL and carbohydrate to form ABS when compared with the remaining ILs reported in the literature, namely [C_4_C_1_im][BF_4_], [C_4_C_1_im][CF_3_SO_3_], and [C_2_C_1_py][C_4_F_9_SO_3_]. This opposite behavior reinforces the high tailoring ability of the investigated cholinium-derived ILs by only changing the size of the aliphatic moieties and number of hydroxyl groups at the cation since they cover the whole hydrophobicity range showed up to date with ILs in ABS formation with carbohydrates.

### ABS as Alternative Strategies for the Simultaneous Separation of Antioxidants and Carbohydrates From Food Waste

Given that in ABS formed by ILs and carbohydrates there is the spontaneous separation of the two phase-forming components above given concentrations, and that previous works on ABS formed by ILs and salts show a preferential partition of antioxidants to the IL-rich phase (Cláudio et al., 2010, 2012, 2014), we hypothesized the application of ABS formed by ILs and carbohydrates as one-step separation strategies of antioxidants and carbohydrates from food waste. After addressing the ILs cytotoxicity and their ability to create ABS, this hypothesis was here evaluated under a perspective of resource efficiency and circular economy. To this end, ABS composed of the three cholinium-derived ILs and two of the less expensive and most abundant carbohydrates (D-glucose and D-sucrose) were investigated to simultaneously separate antioxidants and carbohydrates from an expired commercial vanillin-rich pudding. However, with the IL [N_1(2OH)__(2OH)__(2OH)_][NTf_2_] only D-glucose is able to promote phase separation, and consequently with this IL only ABS formed with D-glucose were evaluated. Based on the phase diagrams discussed above, a common mixture composition at the biphasic region of the several studied ABS was chosen, formed by 25 wt% of D-glucose or D-sucrose + 50 wt% of IL + 25 wt% of aqueous solution of pudding. The representation of this mixture composition is shown in Supplementary Figure S5.

**Figure 5:**
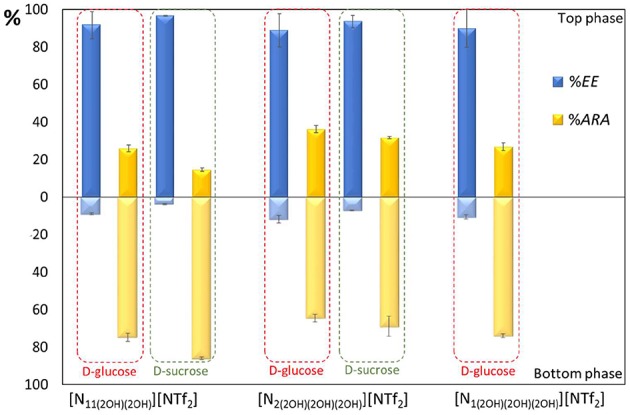
Carbohydrates extraction efficiency (%*EE*) and antioxidant relative activity (%*ARA*) in each phase using ABS composed of 25 wt% D-glucose or D-sucrose + 50 wt% IL + 25 wt% aqueous solution of pudding.

The ABS extraction efficiency (%*EE*) for carbohydrates and antioxidant relative activity (%*ARA*) in each ABS phase were calculated using Equations (1–3), being shown in Figure 5. Detailed data and associated standard deviations are given in Supplementary Table S5. In all systems studied the top phase is mainly composed of carbohydrates and water, with the bottom phase corresponding to the IL-rich phase, as experimentally confirmed by conductivity measurements of both phases. The relative densities of the ABS phases on the studied systems are the opposite of most systems previously reported (Freire et al., 2012) due to the high density of the fluorinated ILs (Pereiro et al., 2013; Neves et al., 2014) used in this work.

In all studied systems, antioxidants majorly partition to the IL-rich phase, while carbohydrates are mainly present in the top phase. The separation of both types of compounds to opposite phases occurs in one-step, with %*EE* of carbohydrates to the carbohydrate-rich phase ranging between 89 and 92% for the systems formed by D-glucose and between 94 and 97% for the systems comprising D-sucrose. The %*ARA* of antioxidants to the IL-rich phase ranges between 65 and 75% in the systems with D-glucose and between 68 and 85% in the systems formed by D-sucrose. Being the pudding vanilla flavored, vanillin is one of the main antioxidants present. Previous studies demonstrated that vanillin preferentially migrates to the IL-rich phase (Cláudio et al., 2010) in ABS formed by ILs and a strong salting-out salt (K_3_PO_4_). Here, a class of weaker salting-out agents is used, namely carbohydrates, yet antioxidants still majorly partition to the IL-rich phase. On the opposite, sugars are preferentially enriched in the top phase, as expected given that these ILs were designed to be able to form ABS with carbohydrates and spontaneously separate at the concentrations used. Given that no significant differences exist in terms of separation performance between the three ILs investigated, we suggest [N_2(2OH)__(2OH)__(2OH)_][NTf_2_] as the most adequate IL to be used since it has a lower cytotoxicity, being this the IL used in the recyclability studies discussed below.

**Figure 6:**
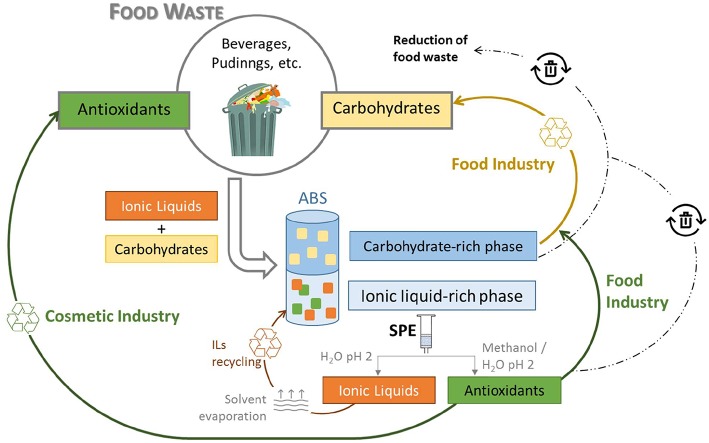
Schematic representation of the proposed strategy for the separation and reuse of carbohydrates and antioxidants obtained from food waste. The IL recycling possibility is also shown.

Previous works employing ABS formed by ILs and carbohydrates focused on the separation of amino acids, alkaloids, terpenoids, and food colorants (Freire et al., 2011; Ferreira et al., 2016). These studies were carried out with model aqueous solutions, and their applicability in the separation of value added compounds from real samples, as carried out in this work, has not been attempted. Furthermore, this work on ABS constituted by ILs and carbohydrates takes advantage of the carbohydrates present and required for ABS formation in the design of a more integrated separation strategy, in which two classes of value added compounds (carbohydrates and antioxidants) are enriched in different phases. This strategy reduces the number of different compounds present in each phase and the number of purification steps. Overall, the investigated ABS composed of ILs and carbohydrates are promising platforms to separate carbohydrates and antioxidants from real food waste. The carbohydrates recovered may be reused by the food industry, while the antioxidants may be reused by the food or cosmetic industries, as summarized in Figure 6. It should be however remarked that although the toxicity of the investigated ILs toward human intestinal cell lines is substantially lower than those reported for other ILs based on fluorinated anions, we cannot conclude if the continuous exposure to these ILs is completely safe before a complete risk assessment is performed.

Foreseeing the described applications of the extracted antioxidants, it is of high significance to separate and recover these compounds from the IL-rich fraction, while improving the sustainable character of the developed separation strategy by attempting the IL recycling. The recyclability of the IL-rich phase was performed for two times using the ABS composed of [N_2(2OH)__(2OH)__(2OH)_][NTf_2_] and D-glucose. After the simultaneous separation of carbohydrates and antioxidants, SPE cartridges were used to separate the antioxidants from the IL-rich phase, allowing the IL recyclability and evaluation of the ABS separation performance for two more times. The use of the recycled IL in new ABS allows to obtain an %*ARA* of antioxidants to the IL-rich phase ranging between 72 and 82% and an %*EE* of carbohydrates to the carbohydrate-rich phase ranging between 89 and 91%. Detailed data are given in Supplementary Figure S7 and Supplementary Table S6. The IL recycling possibility is depicted in Figure 6. In each recycling assay, 85 wt% of the IL was recovered; however, it should be stressed that this value can be increased when working at a large-scale and under optimized conditions of IL recovery and recycling. Overall, these results show that the IL can be recovered and recycled without losses on the ABS separation performance, while allowing the recovery of antioxidants from the IL-rich phase.

## Conclusions

Food waste is an important economic, environmental and social problem that requires innovative solutions in order to reduce its burden. The management of food waste is a priority mitigation measure to reduce emissions intensity and the carbon footprint of the food production chain, which can be achieved through the implementation of new technological solutions such as by transforming food waste into products with marketable value. Accordingly, there is a relevant need to develop sustainable and cost-effective technologies to recover value added compounds from food waste. Based on this need, we here proposed the use of ABS composed of ILs and carbohydrates to simultaneously separate two value added compounds from food waste.

We successfully demonstrated that cholinium-derived ILs, if properly designed, can form ABS with carbohydrates. These new ILs are substantially less toxic than other fluorinated ILs commonly used, as demonstrated by cytotoxicity assays toward the human colon carcinoma cell line (Caco-2). Based on the ability to create ABS with carbohydrates, these systems were finally evaluated as separation platforms of carbohydrates and antioxidants from food waste, namely from a vanilla pudding. The separation of these products occurs in one-step with the studied systems, where carbohydrates are enriched in a carbohydrate-rich phase and antioxidants are mainly present in the IL-rich phase. Extraction efficiencies of carbohydrates ranging between 89 and 97% to the carbohydrate-rich phase, and antioxidant relative activities ranging between 65 and 85% in the IL-rich phase were obtained. Furthermore, antioxidants from the IL-rich phase were recovered by solid-phase extraction, and the IL was recycled for two times with no losses on the ABS separation performance. Given that no significant differences exist in terms of separation performance between the three ILs investigated, [N_2(2OH)__(2OH)__(2OH)_][NTf_2_] is proposed as the most adequate IL to be used since it has a lower cytotoxicity at higher IL concentrations. Overall, the investigated ABS composed of ILs and carbohydrates are promising platforms to simultaneously (one-step) separate carbohydrates and antioxidants from real food waste, in which ILs can be recovered and recycled under a circular economy approach.

## Data Availability

The raw data supporting the conclusions of this manuscript will be made available by the authors, without undue reservation, to any qualified researcher.

## Author Contributions

MGF, JE, JC, and LR conceived and planed the work. PR synthesized and characterized all ILs. MBF, AC, and AS planned the cytotoxicity experiments, carried out the experimental assays, and analyzed the results. MF and CN carried out the experiments on the phase diagrams, and antioxidant activity and carbohydrates quantification assays. All authors contributed to the interpretation of the acquired data and to the manuscript preparation.

### Conflict of Interest Statement

The authors declare that the research was conducted in the absence of any commercial or financial relationships that could be construed as a potential conflict of interest.
