# Supplementary material for: Simultaneous Separation of Antioxidants and Carbohydrates From Food Wastes Using Aqueous Biphasic Systems Formed by Cholinium-Derived Ionic Liquids
Source: Front Chem. 2019 Jun 27;7:459. doi: 10.3389/fchem.2019.00459 (PMC6610481; doi:10.3389/fchem.2019.00459)
Supplement: Supplementary file 1 [file Table_1.pdf]

Supplementary Material

Simultaneous Separation of Antioxidants and Carbohydrates from Food Waste using Aqueous Biphasic Systems formed by Cholinium-derived Ionic Liquids

Catarina M. S. S. Neves[1], Marcos Figueiredo[1], Patrícia M. Reis[2], Ana C. A. Sousa[1], Ana C. Cristóvão[3], Mariana B. Fiadeiro[3], Luís Paulo N. Rebelo[2], João A. P. Coutinho[1], José M. S. S. Esperança[2*], Mara G. Freire[1*]

[1]CICECO – Aveiro Institute of Materials, Department of Chemistry, University of Aveiro, Aveiro, Portugal
[2]LAQV/REQUIMTE, FCT-NOVA, Caparica, Portugal
[3]CICS-UBI – Health Sciences Research Centre, University of Beira Interior, Covilhã, Portugal

*** Correspondence:** Corresponding Author: Dr. José M. S. S. Esperança and Dr. Mara G. Freire (jmesp@fct.unl.pt; maragfreire@ua.pt)

1. Supplementary Figures and Tables 2
1.1 Synthesis of [$N_{11(2OH)(2OH)}$][NTf_2], [$N_{1(2OH)(2OH)(2OH)}$][NTf_2] and [$N_{2(2OH)(2OH)(2OH)}$][NTf_2] 2
1.2 Supplementary Tables....................4
Supplementary Table S1....................4
Supplementary Table S2....................5
Supplementary Table S3....................8
Supplementary Table S4....................11
Supplementary Table S5....................12
Supplementary Table S6....................12
1.3 Supplementary Figures 13
Supplementary Figure S1. 13
Supplementary Figure S2. 14
Supplementary Figure S3. 15
Supplementary Figure S4. 16
Supplementary Figure S5. 17
Supplementary Figure S6. 18
Supplementary Figure S7. 19
2 References 19

1. Supplementary Figures and Tables

1.1 Synthesis of [$N_{11(2OH)(2OH)}$][NTf_2], [$N_{1(2OH)(2OH)(2OH)}$][NTf_2] and [$N_{2(2OH)(2OH)(2OH)}$][NTf_2]

The reagents and solvents used were: lithium bistriflimide (Iolitec, 99 %), sulfuric acid (Merck, 98 %), 2-dimethylaminoethanol (Aldrich, 99 %), N-methyldiethanolamine (Aldrich, 99 %), triethanolamine (Aldrich, 99 %), 2-chloroethanol (Aldrich, 99 %) 1-bromoethane (Fluka, 98 %), hexane (Riedel-de-Häen, 95 %), diethyl ether (Riedel-de-Häen, 99%), ethyl acetate (Riedel-de-Häen, 99 %) and acetone (Riedel-de-Häen, 99 %).

The synthesis of [$N_{11(2OH)(2OH)}$][NTf_2], [$N_{1(2OH)(2OH)(2OH)}$][NTf_2] and [$N_{2(2OH)(2OH)(2OH)}$][NTf_2] starts with the preparation of the homologue halide salt according to scheme:

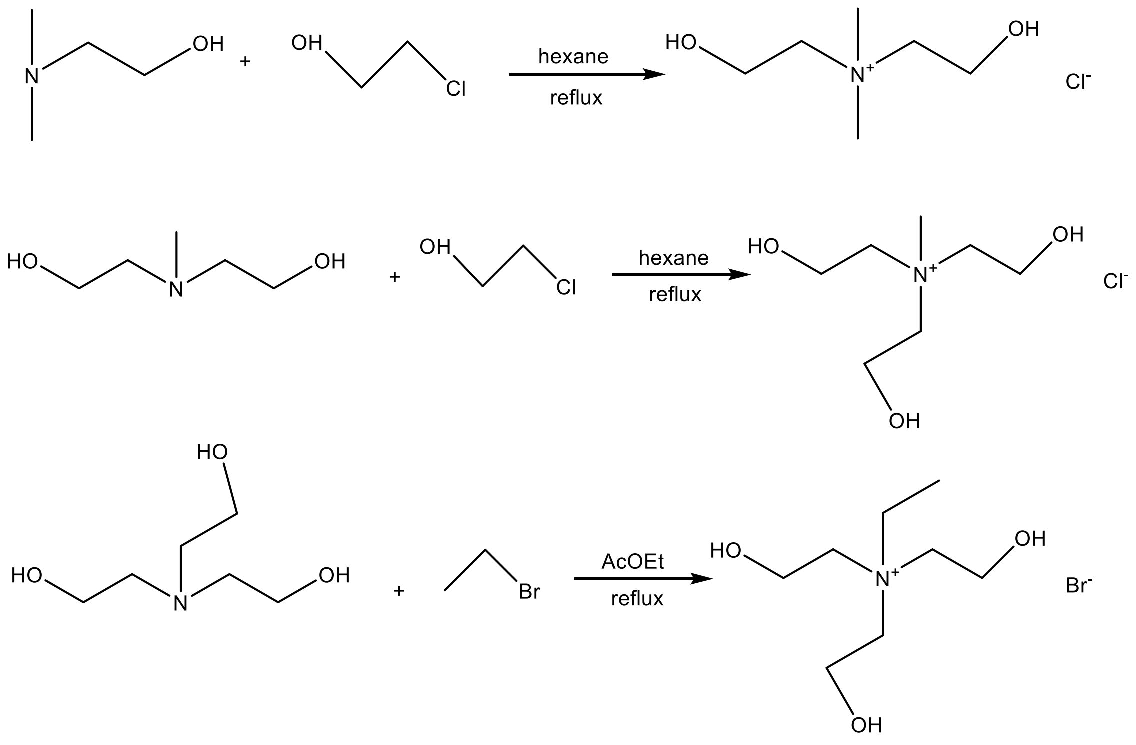

The reaction mixture was warmed up under reflux for one day. The obtained halide salt was washed with acetone and diethyl ether. The solvent is decanted and the final product is dried under vacuum at 50º C during two days.

An aqueous solution of $[N_{11(2OH)(2OH)}]Cl$, $[N_{1(2OH)(2OH)(2OH)}]Cl$ or $[N_{2(2OH)(2OH)(2OH)}]Br$ was passed through an anionic exchange Amberlite resin collumn, to obtain the corresponding hydroxide salt. The hydroxide salt produced was reacted with a solution of $HNTf_2$. The $HNTf_2$ was synthetized in house by adding H_2SO_4 to pure $LiNTf_2$, followed by the distillation of the $HNTf_2$, forming a white powder. The titration of the halide salts with the acid was stopped when the pH of the solution reached 7. The prepared ILs, $[N_{11(2OH)(2OH)}][NTf_2]$, $[N_{1(2OH)(2OH)(2OH)}][NTf_2]$ and $[N_{2(2OH)(2OH)(2OH)}][NTf_2]$, were dried in by evaporation in a rotavapour to remove the large excess of water and finally dried under vacuum at 333 K for at least 2 days.

The $AgNO_3$ test was performed to all ionic liquids to confirm the absence of any halides (chloride or bromide). The water content of the dried ILs, measured by coulometric Karl Fischer titration, was always below 500 ppm.

All ionic liquids were characterized by ^1H and ^{19}F NMR experiments in dmso-d_6 and elemental analysis. Nuclear magnetic resonance (^1H and ^{19}F NMR) spectra were recorded on a Bruker Avance III 400 spectrometer at 400 MHz and 376 MHz, respectively. Chemical shifts are expressed in ppm and *J* values are given in Hz. Proton chemical shifts are reported with the solvent reference (dmso, δ 2.50 ppm). MestreNova software was used for data analysis.

IL $[N_{1(2OH)(2OH)(2OH)}][NTf_2]$

^1H NMR: 3.13 (s, 3H, NC**H₃**), 3.51 (t, 6H, *J* = 6 Hz, $NC\mathbf{H_2}CH_2OH$), 3.83 (m, 6H, $NCH_2C\mathbf{H_2}OH$), 5.24 (t, 3H, *J* = 4 Hz, $NCH_2CH_2O\mathbf{H}$).

^{19}F NMR: -78.71

Elemental analysis calculated (found): %C 24.33 (24.47); %H 4.08 (3.78); %N 6.30 (6.04); %S 14.43 (14.40).

IL $[N_{2(2OH)(2OH)(2OH)}][NTf_2]$

^1H NMR: 1.24 (t, 3H, *J* = 6 Hz, $NCH_2C\mathbf{H_3}$), 3.45-3.52 (m, 8H, $NC\mathbf{H_2}CH_3$ and $NC\mathbf{H_2}CH_2OH$), 3.83 (m, 6H, $NCH_2C\mathbf{H_2}OH$), 5.22 (t, 3H, *J* = 4 Hz, $NCH_2CH_2O\mathbf{H}$).

^{19}F NMR: -78.71

Elemental analysis calculated (found): %C 26.20 (26.16); %H 4.40 (4.47); %N 6.11 (6.14); %S 13.99 (14.20).

IL [$N_{112OH2OH}$][NTf_2]

^{1}H NMR: 3.11 (s, 6H, NC**H_3**), 3.45 (m, 4H, NC**H_2**CH_2OH), 3.84 (m, 4H, NCH_2C**H_2**OH), 5.27 (t, 2H, *J* = 4 Hz, NCH_2CH_2O**H**).

^{19}F NMR: -78.71

Elemental analysis calculated (found): %C 23.19 (23.55); %H 3.89 (3.97); %N 6.76 (7.21); %S 15.48 (15.45).

1.2 Supplementary Tables

Supplementary Table S1.

List of combinations of ILs and carbohydrates tested to form ABS at 25 °C and atmospheric pressure: (×) do not form ABS; (✓) form ABS.

Carbohydrates	[$N_{11(2OH)(2OH)}$][NTf_2]	[$N_{2(2OH)(2OH)(2OH)}$][NTf_2]	[$N_{1(2OH)(2OH)(2OH)}$][NTf_2]
D-glucose	✓	✓	✓
D-mannose	✓	✓	×
D-galactose	✓	×	×
D-fructose	✓	✓	✓
L-arabinose	✓	×	×
D-xylose	✓	✓	×
D-maltose	✓	✓	×
D-sucrose	✓	✓	×
D-maltitol	✓	✓	✓
D-sorbitol	✓	✓	✓
Xylitol	✓	×	×

Supplementary Table S2.

Experimental weight fraction data (*w*) for the systems composed of carbohydrate (CH) + $[N_{11(2OH)(2OH)}][NTf_2]$ + H_2O at 25 °C and atmospheric pressure.

D-maltitol		D-sorbitol		D-glucose		D-maltose	
100 *w*IL	100 *w*CH	100 *w*IL	100 *w*CH	100 *w*IL	100 *w*CH	100 *w*IL	100 *w*CH
39.59	3.50	66.06	2.52	65.26	1.43	66.30	1.30
37.16	3.78	53.65	2.98	62.40	2.00	61.18	1.52
35.47	4.03	49.16	3.59	59.18	2.50	59.51	1.85
34.53	4.39	44.71	4.03	55.84	3.23	57.73	2.15
32.15	4.97	41.79	4.49	52.16	3.46	55.25	2.43
31.02	5.28	37.94	5.39	49.47	4.07	52.83	2.68
30.31	5.54	35.53	5.61	46.30	4.14	50.69	2.96
28.68	6.02	33.66	5.85	45.38	4.50	48.82	3.16
27.54	6.63	32.13	6.09	43.58	4.78	47.69	3.35
26.10	7.37	30.84	6.39	42.39	4.97	46.49	3.59
25.01	7.77	30.13	6.73	40.88	5.11	44.53	4.00
23.82	8.45	28.60	7.39	39.57	5.21	42.98	4.17
23.10	8.82	26.67	7.72	38.69	5.24	41.61	4.33
22.25	9.49	26.27	8.07	37.58	5.59	40.47	4.74
21.74	9.88	25.23	8.64	36.40	5.68	39.03	5.38
20.84	10.66	24.25	9.18	35.56	5.86	37.78	5.49
19.81	11.40	23.48	9.72	34.69	6.18	36.76	5.57
19.07	12.25	22.61	10.13	33.98	6.26	35.61	5.63
18.14	13.18	21.79	10.91	33.30	6.56	34.77	6.02
17.47	13.91	20.49	11.87	32.35	6.96	33.43	6.71
16.70	14.96	19.36	12.88	30.72	7.27	32.56	7.05
16.13	15.60	18.62	13.67	29.71	7.61	31.53	7.27
14.71	17.63	17.86	14.67	28.58	7.82	30.76	7.58
13.52	19.87	16.73	15.86	27.57	8.31	29.18	8.31
11.98	21.58	15.90	16.87	26.56	8.75	28.21	8.87
11.36	22.84	13.83	19.75	25.62	9.39	27.56	9.18
9.28	27.93			24.62	10.00	26.54	9.64
				23.91	10.40	25.60	10.09
				23.11	10.73	24.77	10.62

Xylitol		D-fructose		D-mannose		D-xylose	
100 *w*IL	100 *w*CH	100 *w*IL	100 *w*CH	100 *w*IL	100 *w*CH	100 *w*IL	100 *w*CH
66.55	2.54	58.32	2.43	67.19	1.82	67.84	1.80
58.02	3.15	55.88	3.71	61.50	2.64	63.90	2.55
48.21	3.37	50.22	4.39	56.50	3.32	58.93	3.38
40.92	3.47	46.17	4.96	52.24	3.84	53.31	3.47
36.39	3.66	41.83	5.89	49.30	4.30	50.85	4.07
30.57	4.03	38.82	6.16	46.72	4.78	48.70	4.51
27.25	4.61	37.20	6.75	43.70	5.76	45.38	5.51
23.92	5.37	34.85	7.32	40.61	5.78	41.32	6.05
20.76	6.86	32.80	7.83	37.91	6.16	39.11	6.37
17.09	8.36	31.02	8.37	36.02	6.63	37.51	7.05
14.32	12.86	29.43	8.85	35.19	6.84	35.93	7.29
		27.33	10.13	33.74	7.32	34.84	7.56
		26.19	10.42	33.34	7.38	33.79	8.09
		25.18	10.81	31.95	8.09	32.10	8.52
		23.99	11.45	30.96	8.27	30.54	9.10
		23.06	12.15	30.47	8.49	29.12	9.60
		21.92	12.87	29.46	8.99	27.88	10.30
		20.44	13.79	28.89	9.13	27.12	10.88
		19.65	14.92	28.41	9.36	25.74	11.91
		18.72	16.06	27.17	9.59	24.93	12.32
		17.89	17.05	26.63	9.69	24.19	12.74
		17.27	18.10	25.65	10.86	23.04	13.63
		16.72	18.54	24.78	11.47	21.80	14.81
		15.98	18.97	24.04	11.81	21.28	15.17
		15.11	20.35	23.32	12.24	20.38	15.89
		14.30	21.66	22.82	12.65	19.50	17.07
		13.95	22.19	21.94	13.36	18.69	17.89
		11.99	26.43	21.24	13.93	17.85	18.79
				20.33	14.77	16.08	21.22

D-galactose		L-Arabinose		D-sucrose			
100 *w*IL	100 *w*CH	100 *w*IL	100 *w*CH	100 *w*IL	100 *w*CH	100 *w*IL	100 *w*CH
66.49	1.54	64.93	4.33	68.16	1.21	22.18	12.99
63.80	1.83	51.08	6.05	62.58	1.93	21.77	13.30
61.78	2.26	23.37	13.87	56.93	3.19	21.16	13.93
59.13	2.56	17.38	19.49	50.52	3.42	20.62	14.59
56.81	3.30	38.77	7.76	49.04	3.92	20.05	14.69
54.45	3.52	31.12	9.49	46.33	4.33	19.41	15.25
53.11	3.80	25.41	11.85	45.00	4.85	18.69	16.12
51.26	4.00	18.26	17.52	42.90	5.26	18.02	16.89
49.41	4.20	14.08	22.07	40.38	5.49	17.31	17.79
48.26	4.42			37.96	5.86	16.16	20.19
46.01	4.59			36.85	6.08	15.19	21.88
44.72	4.79			36.20	6.47	13.79	24.56
43.30	4.94			34.62	6.64	12.33	27.56
41.86	5.09			33.89	6.96		
40.68	5.23			32.84	7.16		
40.02	5.37			32.02	7.38		
39.22	5.53			31.08	7.51		
38.38	5.87			30.52	7.77		
37.63	6.02			30.21	8.06		
34.70	6.59			29.32	8.55		
33.85	6.92			28.30	9.05		
32.75	7.19			27.55	9.48		
31.51	7.62			26.67	9.88		
31.18	7.77			25.85	10.22		
30.26	8.00			25.11	10.57		
29.13	8.63			24.76	11.00		
28.32	8.86			23.96	11.53		
27.72	9.12			23.18	12.12		
26.70	9.77			22.67	12.45		

Supplementary Table S3.

Experimental weight fraction data (w) for the systems composed of carbohydrate (CH) + $[N_{2(2OH)(2OH)(2OH)}][NTf_2]$ + H_2O at 25°C and atmospheric pressure.

D-maltitol		D-sorbitol		D-glucose		D-maltose	
100 *w*IL	100 *w*CH	100 *w*IL	100 *w*CH	100 *w*IL	100 *w*CH	100 *w*IL	100 *w*CH
56.76	9.92	74.01	5.84	92.72	1.43	88.55	1.26
55.49	10.37	67.40	5.87	84.98	2.59	79.86	2.26
54.22	10.79	64.96	6.41	80.18	3.74	70.48	5.45
52.23	11.75	63.13	6.96	74.57	4.74	64.91	7.05
51.39	12.30	61.76	7.35	69.33	6.51	48.38	14.25
49.79	12.57	60.45	7.63	64.67	7.21	93.18	1.01
48.30	13.38	59.34	8.16	60.38	8.73	79.12	2.79
46.90	14.01	58.11	8.62	58.14	9.38	72.45	4.67
45.82	14.36	57.12	8.92	54.87	10.75	67.26	6.21
44.23	14.99	55.56	9.29	51.75	11.97	62.84	7.56
43.47	15.30	54.55	9.64	48.94	13.08	55.16	10.65
42.79	15.55	53.24	10.17	46.17	14.24	44.04	15.85
41.41	16.24	52.38	10.50	44.72	14.51		
40.01	16.74	51.09	10.90	42.60	15.32		
38.35	17.55	50.05	11.31	40.63	16.19		
37.35	18.00	48.75	11.77	38.78	16.88		
36.81	18.20	47.41	12.02	37.08	17.61		
35.72	18.62	45.54	13.39	35.47	18.26		
34.96	19.02	44.44	13.69	33.65	19.18		
34.41	19.19	43.69	14.01	31.98	20.04		
33.94	19.33	42.52	14.60	30.50	20.77		
33.11	19.70	41.60	14.82	29.07	21.48		
31.97	20.26	40.23	15.27	27.90	22.03		
30.48	21.20	23.77	22.32	26.68	22.64		
29.88	21.55	20.90	24.23	25.04	23.76		
29.22	21.79	18.54	26.27	23.56	24.73		
28.89	21.95	16.27	28.65	22.14	25.70		
28.36	22.25			20.41	27.11		
27.48	22.72			18.31	28.75		

D-sucrose		Xylitol		D-fructose			
100 *w*IL	100 *w*CH	100 *w*IL	100 *w*CH	100 *w*IL	100 *w*CH	100 *w*IL	100 *w*CH
71.46	15.42	88.97	2.85	92.16	2.29	29.60	28.51
64.89	15.62	77.73	3.75	83.08	4.43	29.22	28.89
61.98	16.32	70.48	5.74	79.21	6.59	28.07	29.72
59.21	17.09	66.18	6.55	75.76	8.46	27.40	29.87
55.48	17.48	60.69	7.98	69.88	11.68	26.32	30.63
53.00	18.38	56.47	9.23	60.99	13.36	25.31	31.18
49.36	19.90	51.32	11.07	58.48	14.38	24.66	31.76
47.49	20.47	48.57	11.57	55.95	15.16	23.64	32.26
45.31	20.96	44.92	12.79	53.55	16.25	22.81	33.00
43.30	21.47	41.85	13.80	51.41	17.30	21.58	34.13
41.46	21.97	39.12	14.65	49.60	18.45	20.20	35.42
38.98	23.02	36.72	15.46	48.06	18.99	18.21	37.40
37.52	23.38	34.56	16.16	46.58	19.64	16.84	39.12
35.54	24.24	33.12	16.50	45.26	20.60		
34.23	24.61	31.33	17.11	43.62	21.35		
33.41	24.86	29.61	17.81	42.10	22.12		
32.47	25.21	28.36	18.29	40.65	22.79		
30.84	26.02	27.00	18.84	39.65	23.35		
29.88	26.27	26.60	19.11	38.61	24.10		
28.50	26.98	26.00	19.50	37.39	24.35		
27.30	27.57	25.19	19.75	36.59	24.93		
26.19	28.19	23.94	20.39	35.77	25.55		
25.15	28.69	22.66	21.05	34.72	25.91		
24.22	29.19	21.29	21.85	33.92	26.33		
23.43	29.76	18.78	23.74	33.09	26.59		
22.60	30.24	14.92	26.78	32.42	27.00		
21.80	30.69			31.75	27.34		
20.81	31.42			31.03	27.78		
19.99	32.08			30.25	28.16		

D-mannose				D-xylose	
100 *w*IL	100 *w*CH	100 *w*IL	100 *w*CH	100 *w*IL	100 *w*CH
87.61	5.30	38.04	23.31	90.97	1.14
78.71	10.44	36.52	24.20	80.68	4.21
70.11	11.05	35.46	24.32	70.75	8.91
66.57	12.10	34.54	24.92	67.15	10.43
64.04	13.18	33.72	25.13	64.71	11.36
61.08	14.06	32.53	25.81	60.96	12.92
59.13	14.98	31.67	25.95	57.99	14.32
56.57	15.80	30.67	26.58	55.06	15.51
54.06	16.41	29.66	27.08	53.71	16.13
52.34	17.10	26.65	28.98	50.45	17.65
49.50	18.61	25.66	29.08	47.34	19.24
48.20	19.25	24.82	29.89	45.24	20.25
46.58	19.79	23.87	30.49	43.33	21.08
45.77	19.99	22.17	31.54	41.69	21.91
44.49	20.54	20.14	33.62	40.17	22.58
43.59	21.24	16.11	37.92	38.19	23.38
42.22	21.66			36.54	24.21
41.18	22.19			29.44	27.91
40.02	22.52			27.59	29.17
39.03	22.90			24.07	31.84

Supplementary Table S4.

Experimental weight fraction data (w) for the systems composed of carbohydrate (CH) + $[N_{1(2OH)(2OH)(2OH)}][NTf_2]$ + H_2O at 25°C and atmospheric pressure.

D-maltitol		D-sorbitol		D-glucose		D-fructose	
100 *w*IL	100 *w*CH	100 *w*IL	100 *w*CH	100 *w*IL	100 *w*CH	100 *w*IL	100 *w*CH
66.30	8.37	88.14	3.34	89.49	2.00	91.86	1.67
38.82	22.52	67.58	8.49	84.30	2.98	80.08	5.51
34.73	25.24	48.75	17.15	79.68	3.76	76.43	7.31
28.78	28.82	46.76	17.75	74.78	5.61	72.82	9.18
25.50	31.27	44.37	19.32	66.65	8.36	69.39	10.84
24.37	32.05	42.08	20.40	57.68	11.61	66.31	12.18
23.66	32.89	40.03	21.56	50.96	15.05	61.77	14.78
21.34	35.05	38.82	21.89	50.72	15.04	55.54	18.14
19.62	36.74	36.30	23.58	48.41	16.33	48.38	23.32
18.36	38.00	33.64	25.07	45.54	17.88	46.98	23.96
17.14	40.01	27.76	28.68	42.59	19.45	44.02	26.17
14.48	43.10	26.86	29.07	42.47	19.68	42.08	27.45
13.50	44.57	25.43	30.25	39.69	21.10	40.25	28.69
12.18	46.16	23.64	31.27	37.08	22.57	38.09	30.31
11.22	49.35	22.36	32.43	35.34	23.46	36.55	31.29
		20.26	34.16	34.41	24.10	35.18	32.22
		17.76	36.89	32.04	25.00	32.28	34.38
		11.27	46.83	29.24	27.48	29.41	36.67
		9.01	49.89	27.02	28.05	28.15	37.72
		5.98	52.98	25.87	29.52	27.32	38.26
		4.95	55.69			26.20	39.13
						24.89	40.34
						22.68	42.31
						21.65	43.31
						19.45	45.46

Supplementary Table S5.

Carbohydrates extraction efficiency (%*EE*) and antioxidant relative activity (%*ARA*) of the phases, with respective standard deviations (σ), using ABS composed of 25 wt% D-glucose / D-sucrose + 50 wt% IL + 25 wt% aqueous solution of pudding.

		D-glucose		D-sucrose	
IL	Phase	%*EE*	%*ARA*	%*EE*	%*ARA*
$[N_{11(2OH)(2OH)}][NTf_2]$	Top	91.69 ± 7.17	24.7 ± 3.5	96.7 ± 0.2	14.8 ± 0.9
	Bottom	8.31 ± 0.01	75.3 ± 2.3	3.3 ± 0.4	85.3 ± 0.6
$[N_{2(2OH)(2OH)(2OH)}][NTf_2]$	Top	88.84 ± 8.88	34.6 ± 6.2	93.5 ± 3.4	31.7 ± 0.7
	Bottom	11.16 ± 0.05	65.4 ± 2.1	6.5 ± 0.2	68.3 ± 5.3
$[N_{1(2OH)(2OH)(2OH)}][NTf_2]$	Top	89.87 ± 0.05	28.7 ± 4.0	-	-
	Bottom	10.13 ± 0.03	71.3 ± 12.1	-	-

Supplementary Table S6.

Carbohydrates extraction efficiency (%*EE*) and antioxidant relative activity (%*ARA*) of the phases, with respective standard deviations (σ), recycling the IL-rich phase three times (3 cycles) of ABS composed of 25 wt% D-glucose + 50 wt% $[N_{2(2OH)(2OH)(2OH)}][NTf_2]$ + 25 wt% aqueous solution of pudding.

		D-glucose	
Cycle	Phase	%*EE*	%*ARA*
1	Top	88.81 ± 8.37	27.7 ± 3.4
	Bottom	11.19 ± 4.85	72.3 ± 7.9
2	Top	90.04 ± 9.10	25.5 ± 3.2
	Bottom	9.96 ± 3.56	74.5 ± 8.1
3	Top	90.69 ± 8.93	17.7 ± 3.0
	Bottom	9.31 ± 4.11	82.3 ± 8.4

1.3 Supplementary Figures

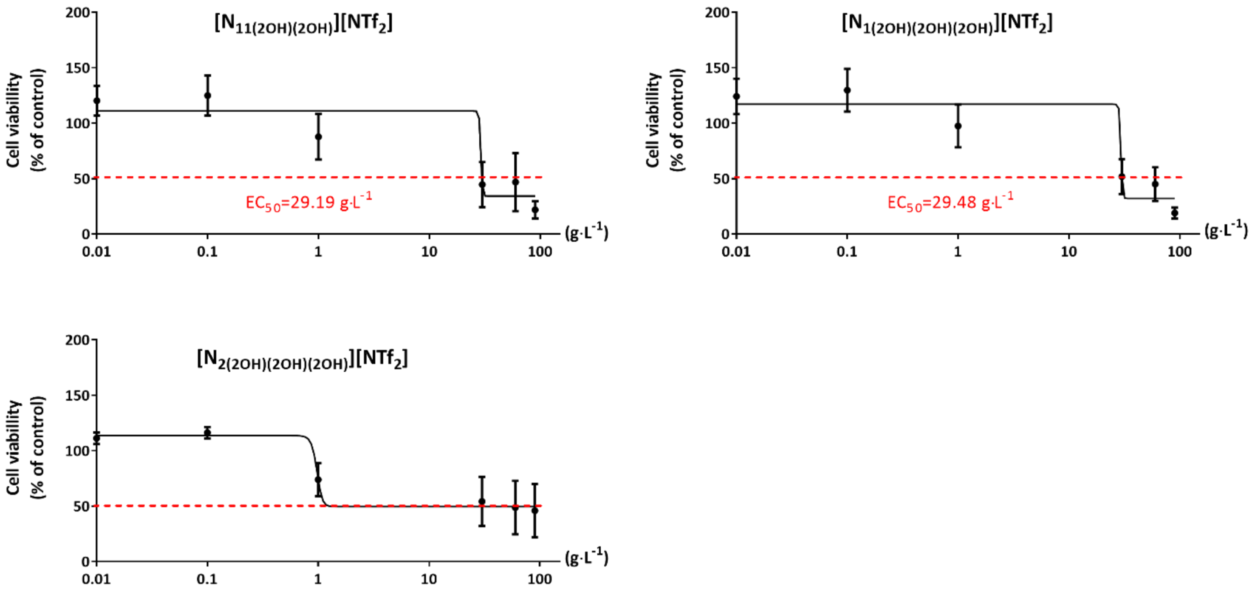

Supplementary Figure S1.

Dose response curves representing Caco-2 cell viability after 24 h exposure to the different ILs. The experimental points correspond to the average of at least four replicates of three independent experiments (*n* = 3). Values of cell viability higher than 100% are indicative of cell proliferation. The curves were fitted using a sigmoidal dose response equation and the automatic outliers fitting method. The dashed line corresponds to the EC_{50}. For $[N_{2(2OH)(2OH)(2OH)}][NTf_2]$ it was not possible to calculate the E_{C50} value.

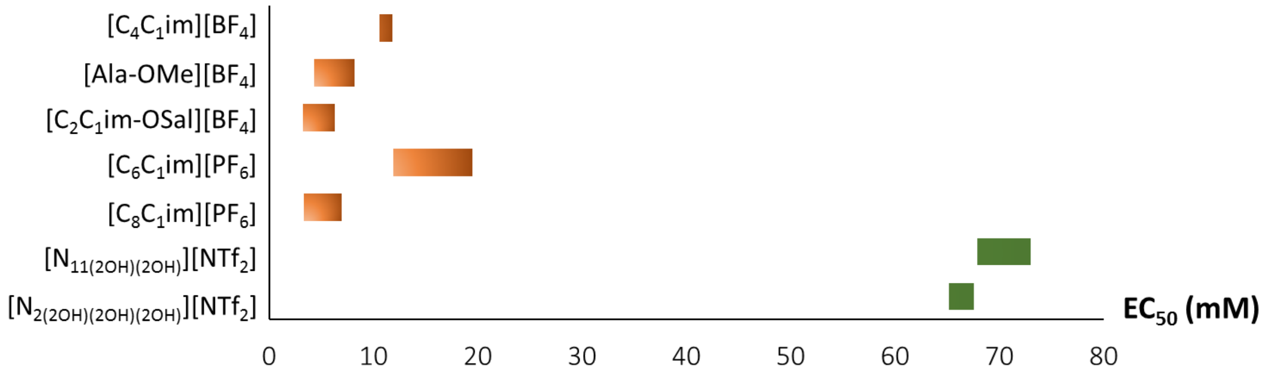

Supplementary Figure S2.

Range of EC_{50} values obtained in this work and in other studies that evaluated the cytotoxic effects of ILs containing fluorinated anions, in Caco-2 cell line after 24 h of exposure. Data for [C_4C_1im][BF_4] (Egorova et al., 2015a), for [Ala-OMe][BF_4] and [C_2C_1im-O][Sal][BF_4] (Egorova et al., 2015b), and for [C_8C_1im][PF_6] and [C_6C_1im][PF_6] (García-Lorenzo et al., 2008).

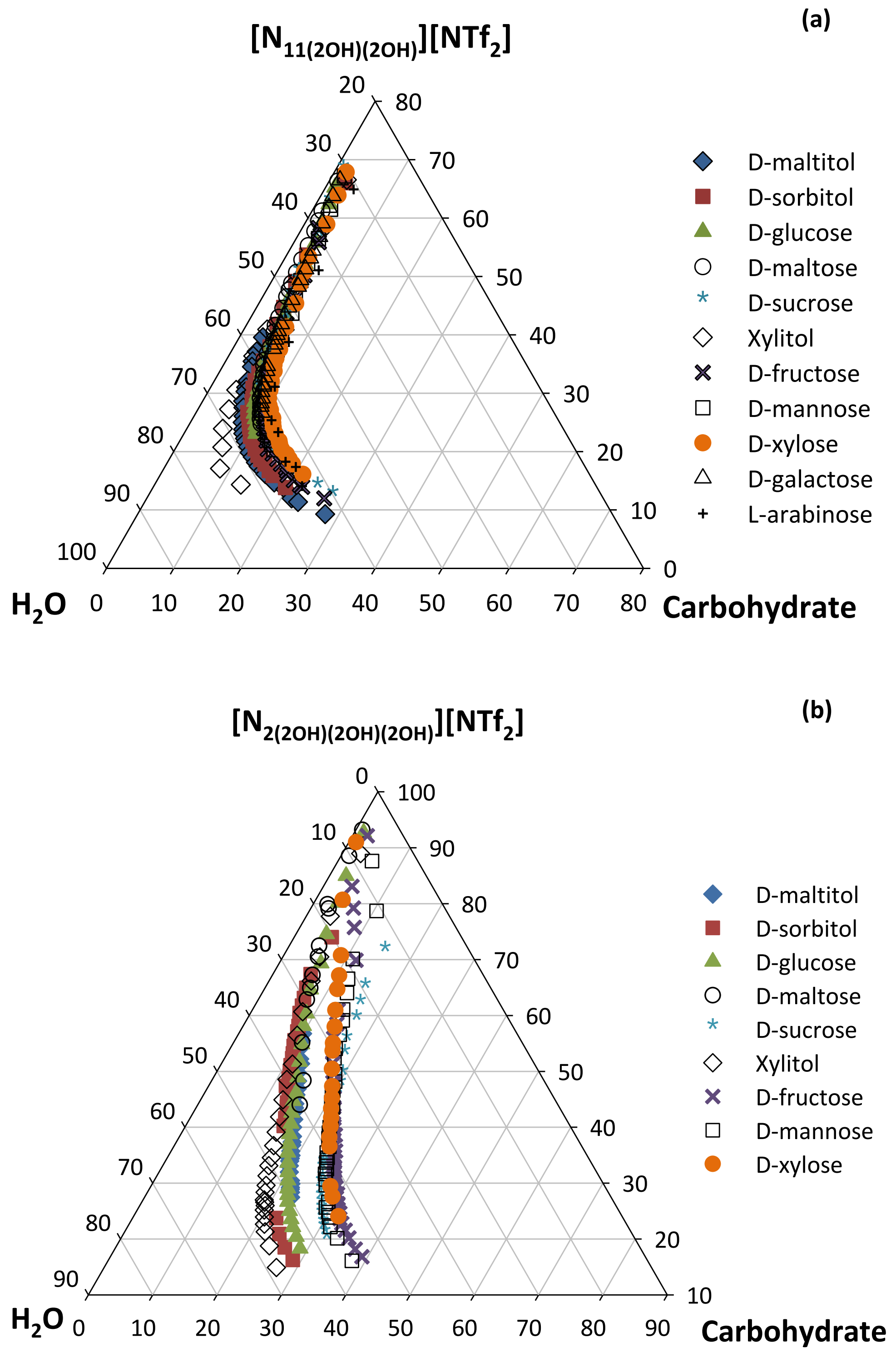

Supplementary Figure S3.

Phase diagrams in weight fraction percentage at 25 ºC and atmospheric pressure for the ABS composed of carbohydrates and the ILs: (a) $[N_{11(2OH)(2OH)}][NTf_2]$ and (b) $[N_{2(2OH)(2OH)(2OH)}][NTf_2]$.

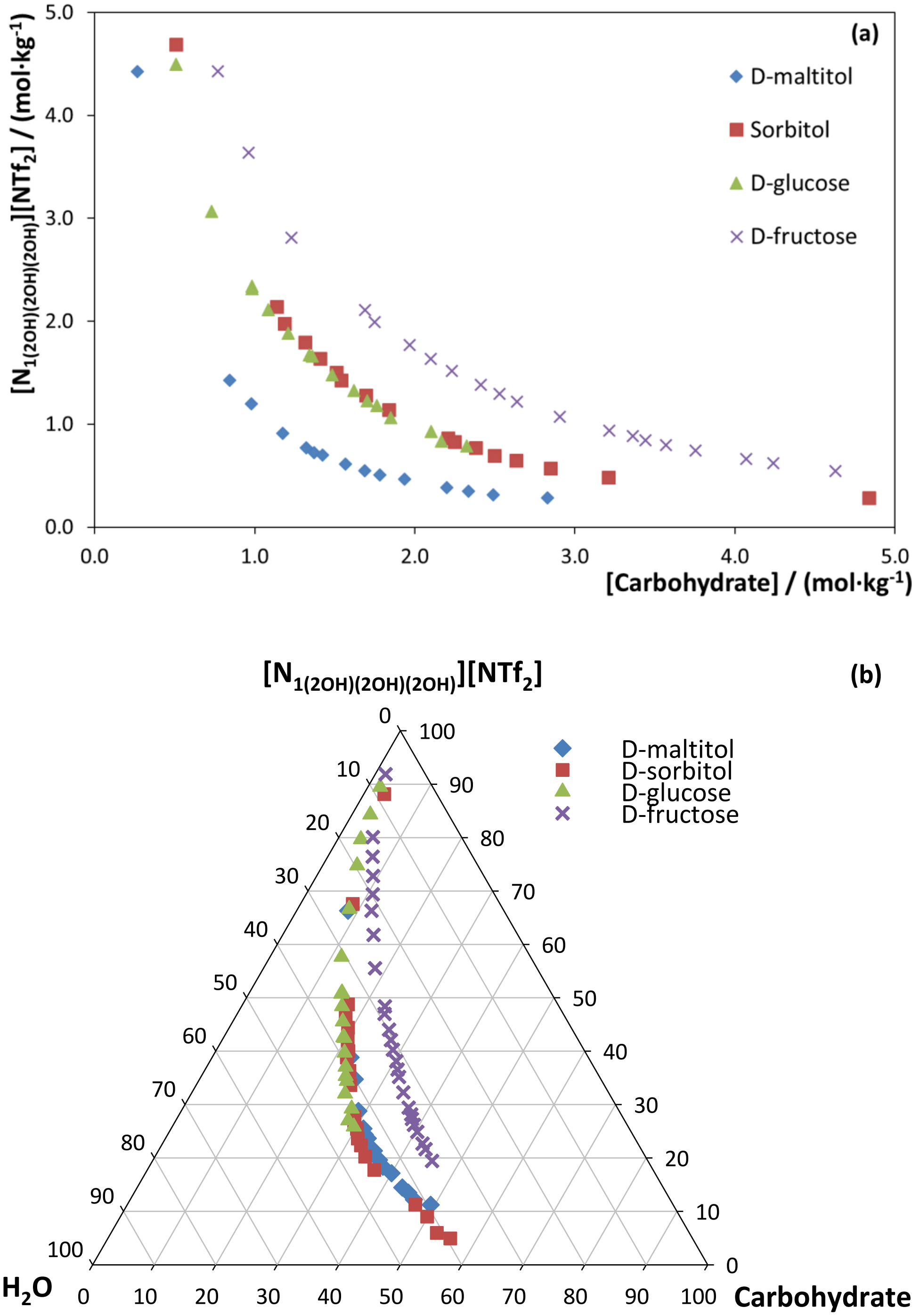

Supplementary Figure S4.

Phase diagrams in molality (a) and mass fraction (b), at 25 ºC and atmospheric pressure, for the ABS composed of different carbohydrates and the IL $[N_{1(2OH)(2OH)(2OH)}][NTf_2]$.

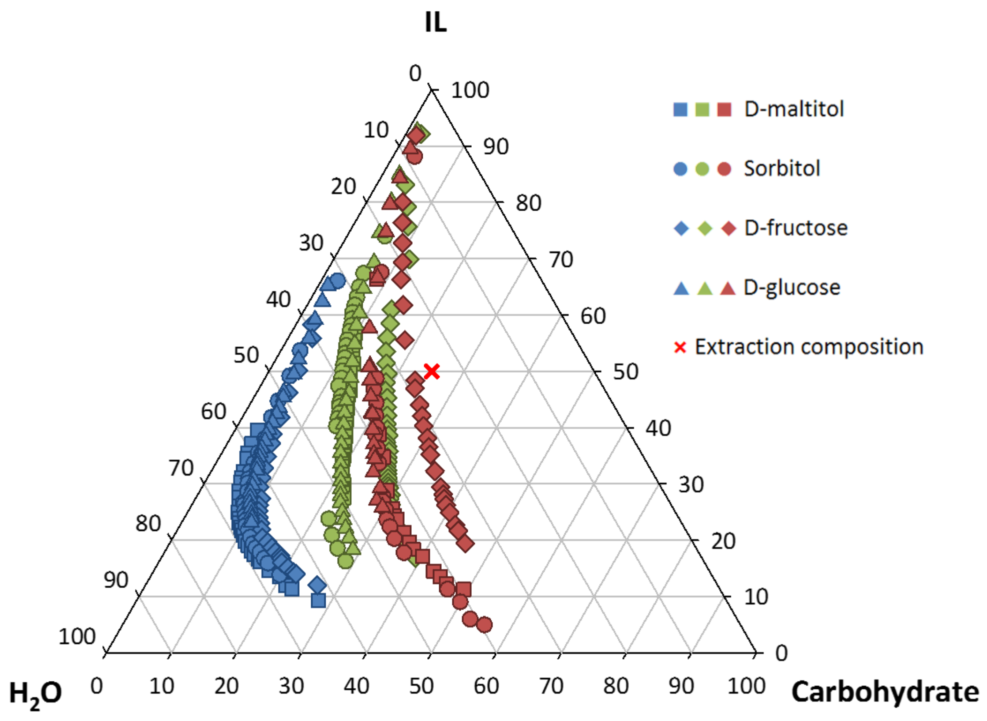

Supplementary Figure S5.

Phase diagrams of ABS composed of water and different carbohydrates and ILs, at 25 ºC and atmospheric pressure, to address the IL effect. Red symbols correspond to the IL $[N_{1(2OH)(2OH)(2OH)}][NTf_2]$; green symbols correspond to $[N_{2(2OH)(2OH)(2OH)}][NTf_2]$; and blue symbols correspond to $[N_{11(2OH)(2OH)}][NTf_2]$.

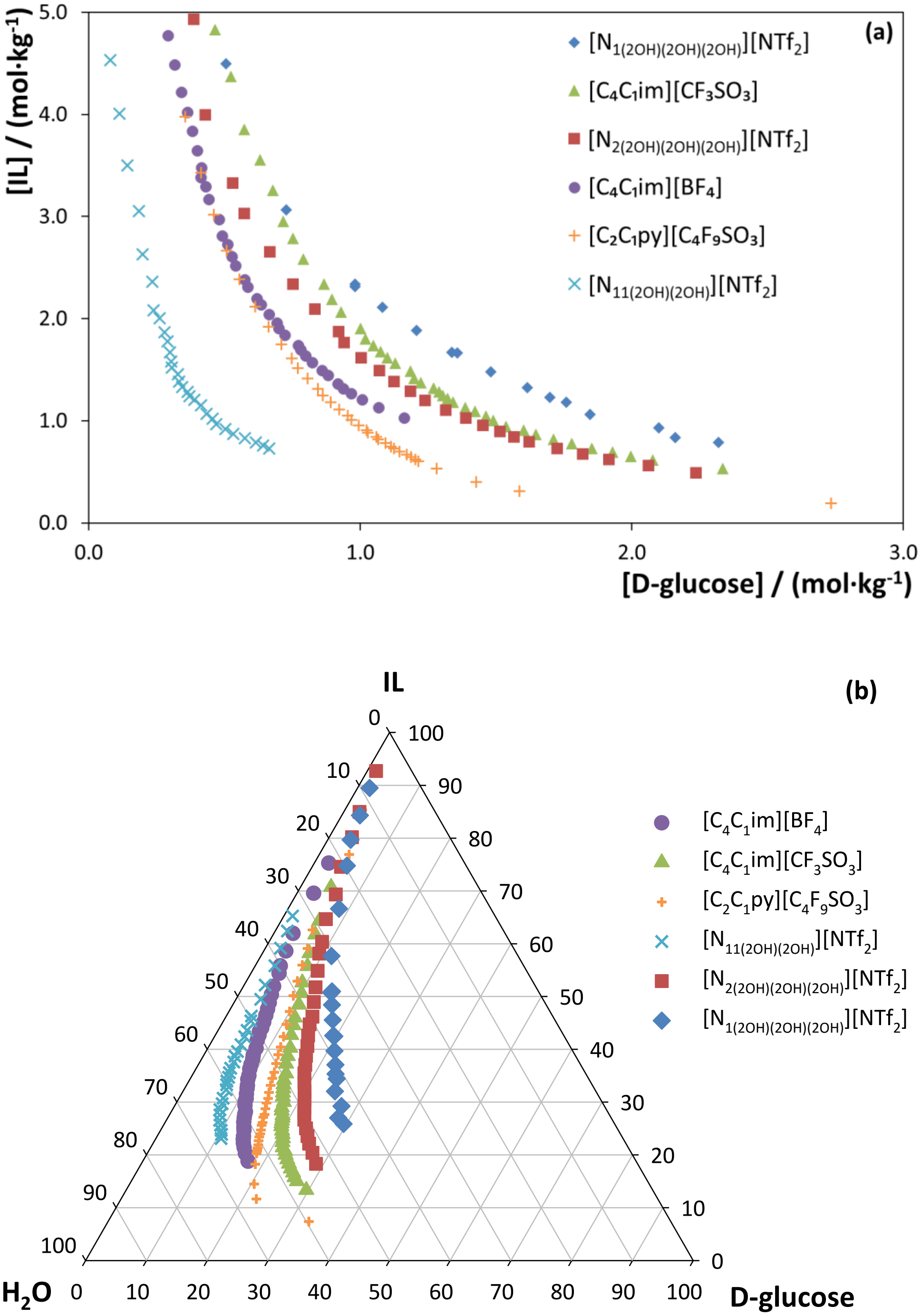

Supplementary Figure S6.

Comparison of phase diagrams of ABS composed of water and D-glucose and different ILs, at 25 ºC and atmospheric pressure, taken from literature: 1-butyl-3-methylimidazolium triflate, $[C_4C_1im][CF_3SO_3]$ (Freire et al., 2011); 1-butyl-3-methylimidazolium tetrafluoroborate, $[C_4C_1im][BF_4]$ (Freire et al., 2011); 1-ethyl-3-methylpyridinium perfluorobutanesulfonate, $[C_2C_1py][C_4F_9SO_3]$ (Ferreira et al., 2016).

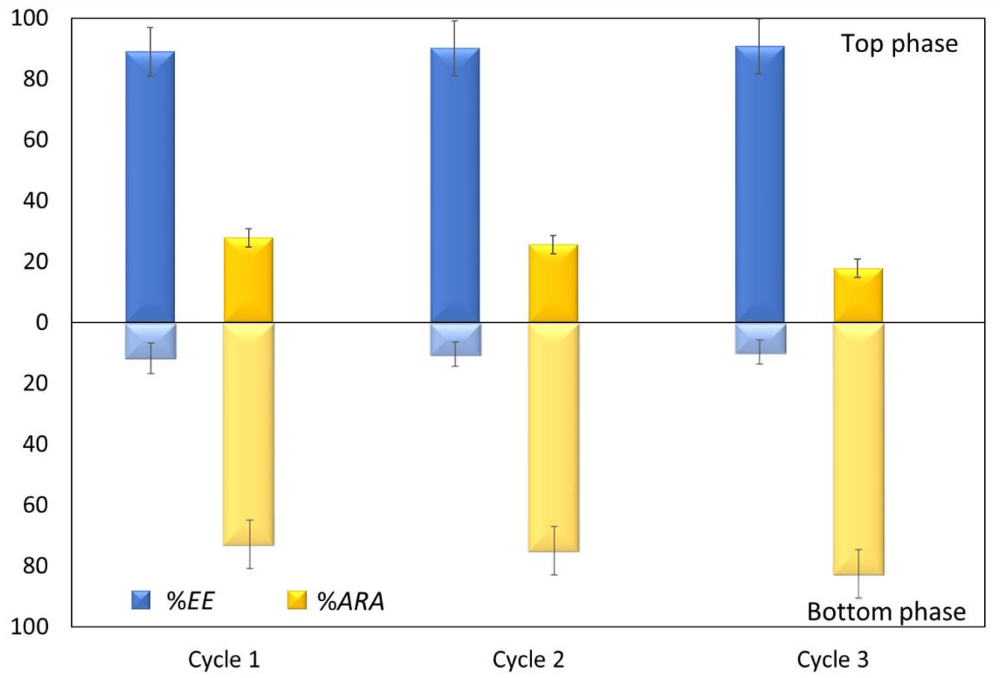

Supplementary Figure S7.

Carbohydrates extraction efficiency (%*EE*) and antioxidant relative activity (%*ARA*) in each phase using ABS composed of 25 wt% D-glucose + 50 wt% [$N_{2(2OH)(2OH)(2OH)}$][NTf_2] + 25 wt% aqueous solution of pudding in three cycles of extraction by recycling the employed IL.

2 References

Egorova, K.S., Seitkalieva, M.M., Posvyatenko, A.V., and Ananikov, V.P. (2015a). An unexpected increase of toxicity of amino acid-containing ionic liquids. *Toxicology Research* 4(1)**,** 152-159. doi: 10.1039/C4TX00079J.

Egorova, K.S., Seitkalieva, M.M., Posvyatenko, A.V., Khrustalev, V.N., and Ananikov, V.P. (2015b). Cytotoxic Activity of Salicylic Acid-Containing Drug Models with Ionic and Covalent Binding. *ACS Medicinal Chemistry Letters* 6(11)**,** 1099-1104. doi: 10.1021/acsmedchemlett.5b00258.

Ferreira, A.M., Esteves, P.D.O., Boal-Palheiros, I., Pereiro, A.B., Rebelo, L.P.N., and Freire, M.G. (2016). Enhanced tunability afforded by aqueous biphasic systems formed by fluorinated ionic liquids and carbohydrates. *Green Chemistry* 18(4)**,** 1070-1079. doi: 10.1039/c5gc01610j.

Freire, M.G., Louros, C.L.S., Rebelo, L.P.N., and Coutinho, J.A.P. (2011). Aqueous biphasic systems composed of a water-stable ionic liquid + carbohydrates and their applications. *Green Chemistry* 13(6)**,** 1536–1545.

García-Lorenzo, A., Tojo, E., Tojo, J., Teijeira, M., Rodríguez-Berrocal, F.J., González, M.P., et al. (2008). Cytotoxicity of selected imidazolium-derived ionic liquids in the human Caco-2 cell

line. Sub-structural toxicological interpretation through a QSAR study. *Green Chemistry* 10(5)**,** 508-516. doi: 10.1039/B718860A.
